# Asymmetric cytokinin signaling opposes gravitropism in roots

**DOI:** 10.1111/jipb.12929

**Published:** 2020-04-24

**Authors:** Sascha Waidmann, Jürgen Kleine‐Vehn

**Affiliations:** ^1^ Department of Applied Genetics and Cell Biology University of Natural Resources and Life Sciences (BOKU) Muthgasse 18 Vienna 1190 Austria

**Keywords:** root system architecture, primary root, lateral root, plant hormones

## Abstract

Plants depend on gravity to provide the constant landmark for downward root growth and upward shoot growth. The phytohormone auxin and its cell‐to‐cell transport machinery are central determinants ensuring gravitropic growth. Statolith sedimentation toward gravity is sensed in specialized cells. This positional cue is translated into the polar distribution of PIN auxin efflux carriers at the plasma membrane, leading to asymmetric auxin distribution and consequently, differential growth and organ bending. While we have started to understand the general principles of how primary organs execute gravitropism, we currently lack basic understanding of how lateral plant organs can defy gravitropic responses. Here we briefly review the establishment of the oblique gravitropic set point angle in lateral roots and particularly discuss the emerging role of asymmetric cytokinin signaling as a central anti‐gravitropic signal. Differential cytokinin signaling is co‐opted in gravitropic lateral and hydrotropic primary roots to counterbalance gravitropic root growth.

In contrast to primary roots, lateral roots partially repress gravitropic growth and establish a distinct gravitropic set point angle (GSA; Digby and Firn 1995). The distinct GSAs of primary and lateral roots ultimately allow the root system to radially explore its surroundings (Mullen and Hangarter 2003). Lateral roots emerge in a 90° angle from the primary root (so‐called stage I; Figure 1). Shortly after emergence, the columella cells of these lateral roots develop statoliths, which are dense amyloplasts. These starch‐filled organelles sediment along the gravity vector to the lateral side of the cell, which induces a partial polarization of PIN3 to this side, inducing asymmetric auxin redistribution and, consequently, differential growth repression at the lower root flank (Rosquete et al. 2013, 2018). It is currently largely unknown what links the statolith sedimentation with cellular polarization. However, this process depends on DEEPER ROOTING 1 (DRO1)/NEGATIVE GRAVITROPIC RESPONSE OF ROOTS (NGR)/LAZY (LZY) genes, which determine whether PINs are polarized toward or away from the site of statolith sedimentation (Uga et al. 2013; Ge and Chen 2016; Guseman et al. 2017; Taniguchi et al. 2017; Yoshihara and Spalding 2017; Furutani et al. 2020).

**Figure 1:**
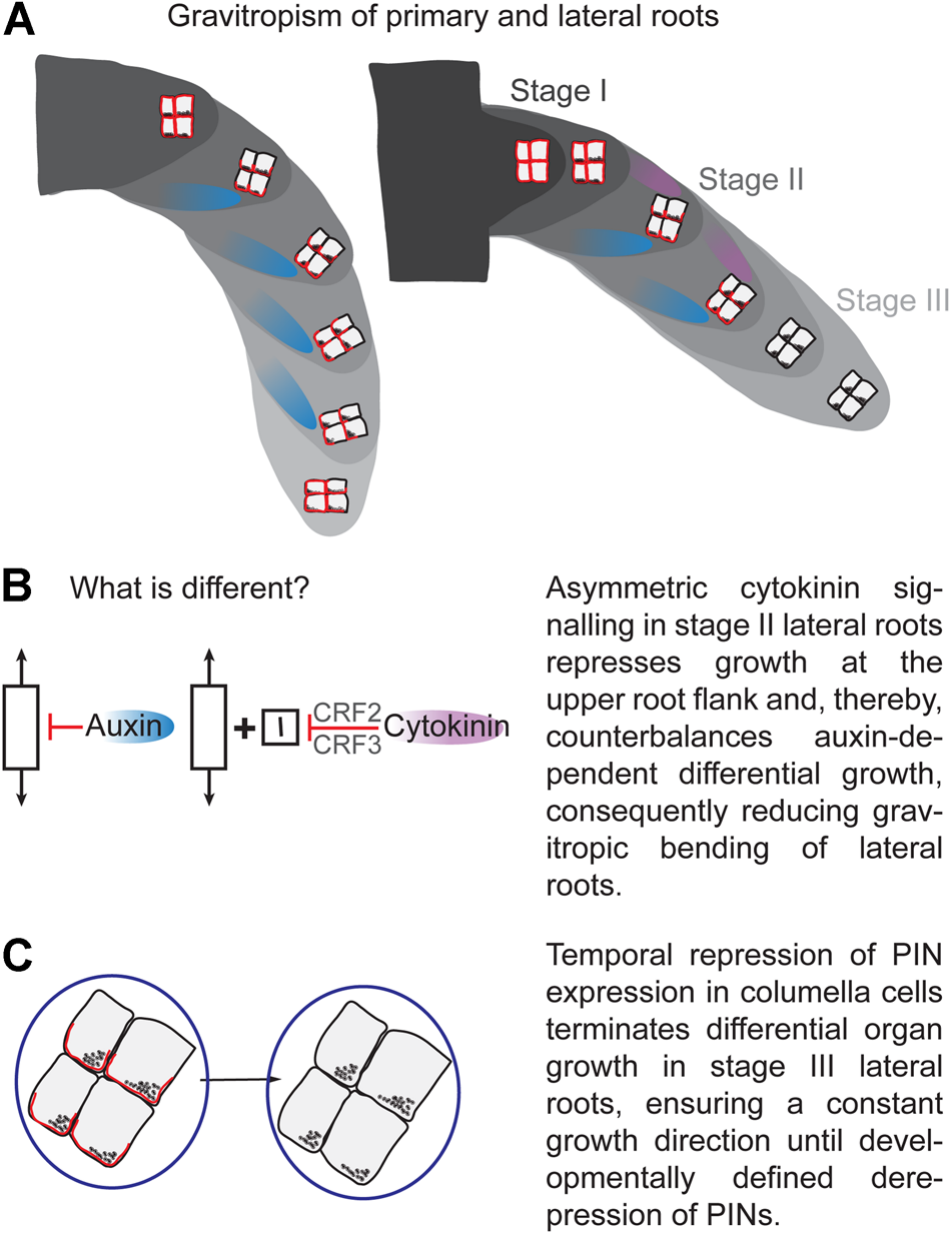
**Gravitropism of primary and lateral roots** (**A**) Schematic depiction of gravitropic growth of the primary (left panel) and lateral root (right panel). Gravity sensing occurs primarily in the central columella cells by sedimentation of statoliths (black dots). In primary roots, PIN3, PIN4, and PIN7 proteins (red lines) are partially polarized toward the side of sedimentation, resulting in an asymmetric auxin signaling and differential growth inhibition at the lower flank of the root (blue area). Lateral roots emerge in a 90° angle from the primary root (stage I) and shortly afterward develop statoliths (black dots) in columella cells. In stage II, PIN4 and PIN7 are repressed and only PIN3 proteins are polarly distributed in the columella cells, resulting in an asymmetric auxin signaling (blue circle) and differential growth inhibition at the lower organ flank. On the other hand, asymmetric cytokinin signaling (purple area) interferes with growth (highlighted in **B**) at the upper side of the lateral root, resulting in reduced organ bending to gravity. Stage III lateral roots are temporally devoid of columella PINs (highlighted in **C**), leading to symmetric auxin signaling and, hence, temporal maintenance of the previously acquired growth direction.

As mentioned earlier, lateral roots show only a partial bending to gravity, establishing a GSA of approximately 62° in the *Arabidopsis* reference accession *Col‐0* (Rosquete et al. 2013; Waidmann et al. 2019). Until recently, the mechanism of how lateral root organs partially suppress gravitropic growth remained enigmatic. Compared to the primary root, several molecular players display distinct expression/activity in lateral roots. In primary roots, PIN3, PIN4, and PIN7 are constantly expressed in columella cells and redundantly control gravitropic root growth (Kleine‐Vehn et al. 2008). These “columella” PINs seem to play functionally divergent roles in lateral roots; only PIN3, but not PIN4 or PIN7, are present in columella cells of young stage II lateral roots (Guyomarc'h et al. 2012; Rosquete et al. 2013). Moreover, the expression of PIN3 is only transient and its repression in stage III lateral roots correlates with non‐differential growth (Figure 1) and, hence, maintenance of GSA in these lateral organs (Guyomarc'h et al. 2012; Rosquete et al. 2013, 2018). Transcription factors FOUR LIPS and MYB88 define PIN3 expression in columella cells of lateral roots (Wang et al. 2015), but the nature of its transient regulation is unknown.

While PIN7 is transcriptionally repressed, PIN4 shows a post‐translational downregulation in stage II columella cells (Rosquete et al. 2013), which might relate to reduced exocyst‐dependent trafficking to the plasma membrane (Ogura et al. 2019). Delivery of PIN4 to the plasma membrane depends on EXOCYST70A3 (EXO70A3), and an increase in PIN4 exocytosis in columella cells leads to more gravitropic growth (Ogura et al. 2019). This agrees with previous assumptions that the partial suppression of PIN4 and/or PIN7 result in comparably weaker redistribution of auxin and consequently reduced gravitropic bending when compared to primary roots (Rosquete et al. 2013).

De‐repression of PIN7 in older (stage IV) lateral roots preferentially terminates radial root expansion by enhancing gravitropic growth in lateral roots (Rosquete et al. 2018). These reports demonstrate that PIN3, PIN4, and PIN7 display a more complex regulation in lateral when compared to primary roots, which determines lateral root organ identity and root system depth.

While stage III lateral roots are temporally devoid of columella PINs, they are perfectly capable of responding to gravity (Mullen and Hangarter 2003; Rosquete et al. 2013, 2018). Reorientation experiments show that gravitropic stimuli de‐represses PIN3, PIN4, and/or PIN7 in stage III lateral roots, allowing the lateral roots to establish asymmetric auxin signaling and to re‐establish its acquired GSA (Rosquete et al. 2013). It still remains largely unknown how lateral roots maintain their acquired GSA and what defines overall lateral root identity (Rosquete et al. 2013; Roychoudhry et al. 2013). It has been envisioned that gravitropic and anti‐gravitropic signals define the oblique directional growth in lateral roots (Roychoudhry et al. 2013). Recently, the phytohormone cytokinin was shown to function as an asymmetric, anti‐gravitropic signal, allowing lateral roots to disregard auxin‐dependent gravitropic growth (Waidmann et al. 2019). Cytokinin and auxin have widespread antagonistic roles in plant development (Schaller et al. 2015), but most intriguingly, auxin and cytokinin display opposing asymmetric signaling at the lower and upper lateral root flank, respectively (Figure 1). Cytokinin interferes with cellular expansion but plays a most prominent role in modulating cell proliferation and differentiation in a cell type‐dependent manner. In shoots, cytokinin preferentially induces cell division, while it interferes with cell cycle progression and initiates differentiation in the primary root (Dello Ioio et al. 2008; Růžička et al. 2009; Schaller et al. 2015). In lateral roots, CYTOKININ RESPONSE FACTOR (CRF) 2 and CRF3 interfere with cell cycle regulators, such as CYCLIN‐DEPENDENT KINASE B1;1 (CDKB1;1), and reduce cell proliferation at the upper organ flank (Waidmann et al. 2019). Consequently, the cytokinin‐induced growth reduction at the upper side indirectly counterbalances the auxin‐induced growth repression at the lower root flank. Accordingly, auxin provides gravitropic input in primary and lateral roots, but cytokinin induces a lateral root‐specific anti‐gravitropic signal, suppressing gravitropic organ bending and allowing radial expansion of the root system (Figure 1).

Unlike in lateral roots, interference with cytokinin signaling affects the levels and localization of auxin transporters in the primary root, generally affecting growth, including gravitropism (Aloni et al. 2004; Růžička et al. 2009; Pernisova et al. 2016). Gravitropic primary roots do not display asymmetric cytokinin signaling based on the synthetic TCSn cytokinin reporter (Waidmann et al. 2019). However, gravitropic main roots may display a variable, asymmetric *ARABIDOPSIS RESPONSE REGULATOR5* (*ARR5)* expression, which does not oppose asymmetric auxin signaling, but presumably coincides with increased auxin levels at the lower root flank (Aloni et al. 2004). However, a recent study reveals a reminiscent spatially defined cytokinin response when primary roots defy gravity during hydrotropism (Chang et al. 2019). Differences in water potential can repress gravitropism of primary roots and redirect root growth toward the water source, which is defined as hydrotropism (Fromm 2019). Accordingly, asymmetric signaling of the phytohormone cytokinin does not only allow lateral roots to disregard gravity (Waidmann et al. 2019) but also reprograms primary root growth during their quest for moisture (Chang et al. 2019). However, the underlying cellular mechanism deviates in gravitropic lateral and hydrotropic primary roots. While cytokinin signaling opposes auxin at the opposite root flank in lateral roots (Waidmann et al. 2019), asymmetric auxin and cytokinin signaling coincide at the side of lower water potential in primary roots (Chang et al. 2019). In addition, asymmetric cytokinin signaling interferes with cell proliferation in lateral roots, but conversely, it induces cell division at the site of lower water potential (Chang et al. 2019; Figure 2). This could be related to the asymmetric induction of *ARR16* and *ARR17*, which are type A ARRs and presumably function as negative regulators of cytokinin (To et al. 2004). The induction of asymmetric *ARR16* or *ARR17* expression is sufficient to induce differential cell division and primary root bending (Chang et al. 2019). Similarly, the asymmetric application of low (1 nmol/L of zeatin) doses of cytokinin phenocopies hydrotropic root growth, differentially inducing cell division and consequently organ bending away from the cytokinin source. In contrast, higher cytokinin levels (100 nmol/L zeatin) repress cell division in primary roots (Chang et al. 2019), proposing not a context‐specific, but a concentration‐dependent effect of cytokinin on primary root growth.

**Figure 2:**
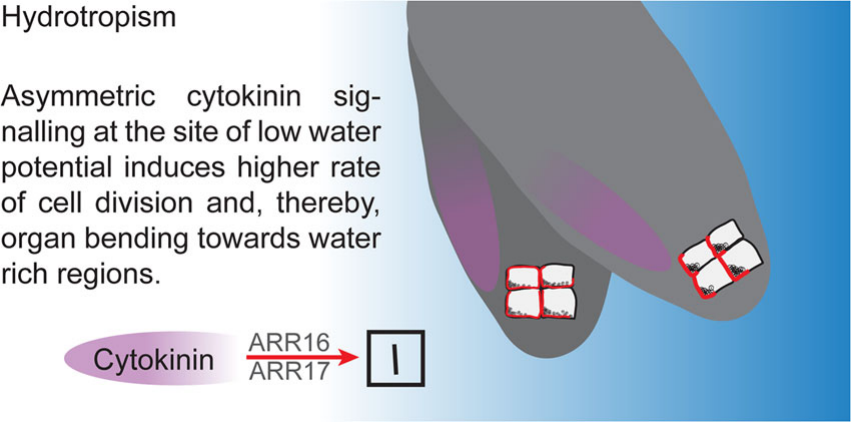
**Hydrotropism in primary roots** Differences in water potential can repress gravitropism of primary roots and redirect root growth toward the water source. Gravity sensing occurs primarily in the central columella cells by sedimentation of statoliths (black dots). In primary roots, PIN3, PIN4 and PIN7 proteins (red lines) are partially polarized toward the side of sedimentation. Asymmetric cytokinin (purple area) and auxin signaling coincide at the side of low water potential. Cytokinin‐induced expression of ARABIDOPSIS RESPONSE REGULATOR (*ARR) 16* and *ARR17* is sufficient to induce asymmetric cell division (inset), resulting in differential growth and organ bending toward the water source.

While the underlying hormonal crosstalk mechanism (non‐overlapping versus overlapping auxin and cytokinin signaling) and resulting cellular responses (cytokinin‐induced repression versus promotion of cell division) strongly deviate in lateral and primary roots, these recent studies illustrate that asymmetric cytokinin signaling is co‐opted in primary (Figure 2) and lateral roots (Figure 1) to defy gravitropic growth. Accordingly, cytokinin emerges as a central anti‐gravitropic determinant, enabling the root system architecture to radially expand and to optimize its growth toward moisture.

## AUTHOR CONTRIBUTIONS

S.W. and J.K.‐V. conceived and wrote the review. All authors read and approved its content.
